# Fast diagnostic test for the identification of an increased genetic predisposition to colon cancer (exemplified on a DNA test for recurrent mutations of the gene MMR)

**DOI:** 10.1186/1897-4287-10-S1-A13

**Published:** 2012-01-12

**Authors:** G Kurzawski, D Dymerska, J Suchy, T Dębniak, J Lubiński

**Affiliations:** 1 International Hereditary Cancer Center, Pomeranian Medical University, Szczecin, Poland

## 

Hereditary nonpolyposis colorectal cancer (Lynch Syndrome, LS) is a genetic disorder, where family members are at high risk of developing cancer of the colon, endometrium, small intestine and urinary tract. The cause for LS is due to constitutional mutations in several mismatch repair genes (MMR) mainly in *MLH1*, *MSH2* and *MSH6.*

The Polish population has been well characterized by a large study comprising ~1000 LS-suspected families. Most nucleotide substitutions (about 90%) were discovered mainly by DHPLC (*denaturing high-performance liquid chromatography*) or sequencing, whereas deletion of one or more exons (about 10% of all mutations) were detected mainly by MLPA (*multiplex ligation-dependent probe amplification*) [1,2].

Over 60% families of the former study, which were affected by recurrent MMR mutations, were taken as a basis to design the following iPLEX/TaqMan test, that allows simultaneous testing of almost all recurrent mutations in only analysis [3] .

The drawback of this kind of analysis are the high costs of the machines (*Sequenom*), for which a reasonable cost-effectiveness is achieved only for large series of probes. Thus the approach is convenient only for high-throughput laboratories.

However the present approach is cost-effective even for testing individual patients. The present test, based on a Taqman PCR analysis, allows a fast identification of the 20 most frequent mutations of the genes *MLH1* and *MSH2*. An analogous test for recurrent mutations of the gene APC could, in a similar way, accelerate the molecular diagnostic of predisposition to FAP.
